# Differences in rates of severe perineal trauma between midwife-led and obstetrician-led care in the Netherlands: A nationwide cohort study

**DOI:** 10.1016/j.heliyon.2024.e24609

**Published:** 2024-01-12

**Authors:** Anna E. Seijmonsbergen-Schermers, Kelly MCM. Peerdeman, Thomas van den Akker, Linde ML. Titulaer, Jan-Paul Roovers, Lilian L. Peters, Corine J. Verhoeven, Ank de Jonge

**Affiliations:** a Amsterdam UMC Location Vrije Universiteit Amsterdam, Midwifery Science, De Boelelaan 1117, Amsterdam, Netherlands; b Midwifery Academy Amsterdam Groningen, Inholland, Amsterdam, Netherlands; c Amsterdam Public Health, Quality of Care, Amsterdam, Netherlands; d University of Groningen, University Medical Center Groningen, Department of General Practice & Elderly Care Medicine, PO Box 196, 9700, AD, Groningen, Netherlands; e Department of Obstetrics and Gynaecology, Leiden University Medical Center, Albinusdreef 2, 2333, ZA, Leiden, Netherlands; f Athena Institute, Vrije Universiteit Amsterdam, De Boelelaan 1085, 1081, HV, Amsterdam, Netherlands; g Department of Obstetrics and Gynaecology, Amsterdam UMC Location AMC, Amsterdam, Netherlands; h Department of Obstetrics and Gynaecology, Maxima Medical Centre, Veldhoven, Netherlands; i Division of Midwifery, School of Health Sciences, University of Nottingham, Nottingham, United Kingdom; j Amsterdam Reproduction and Development, Amsterdam, Netherlands; k School of Nursing and Midwifery, Western Sydney University, Penrith South, New South Wales, Australia

**Keywords:** Obstetric anal sphincter injury, OASI, Severe perineal trauma, SPT, Risk factors, Interventions, Care providers, Place of birth, Maternity care setting

## Abstract

**Objective:**

To investigate trends and rates of severe perineal trauma (SPT), also known as obstetric anal sphincter injury (OASI), between midwife-led and obstetrician-led care in the Netherlands, and factors associated with SPT.

**Methods:**

This nationwide cohort study included registry data from 2000 to 2019 (n = 2,169,950) of spontaneous vaginal births of term, live, cephalic, single infants, without a (previous) caesarean section or assisted vaginal birth.

First, trends of SPT and episiotomy were shown. Second, differences in SPT rates between midwife- and obstetrician-led care were assessed. Third, associations of care factors with SPT were examined. Multivariable logistic regression analyses were used to determine which factors were important in the associations. All outcomes were stratified for parity.

**Results:**

Over time, the SPT incidence increased mainly in midwife-led care and episiotomy rates decreased. Compared to midwife-led care, SPT rates were lower in obstetrician-led care among primiparous women (aOR 0.78; 99 % CI 0.74–0.81) and comparable among multiparous women (aOR 1.04; 99 % CI 0.99–1.10). Among women without epidural analgesia, these differences were smaller for primiparous women (aOR 0.88; 99 % CI 0.84–0.92), but the SPT rate was higher in obstetrician-led care among multiparous women (aOR 1.09; 99 % CI 1.03–1.15). Among women without shoulder dystocia, induction, augmentation, and pain medication, SPT rates were comparable among primiparous women, but higher among multiparous women in obstetrician-led care. In midwife-led care, SPT occurred more often among hospital versus home births. In obstetrician-led care, lower SPT incidences were found among births with epidural analgesia and for multiparous women with induction or augmentation.

**Conclusions:**

Among spontaneous vaginal births, induction, augmentation, and epidural analgesia in obstetrician-led care may be an explanatory factor for the higher incidence of SPT among primiparous women in midwife-led care. More research is needed to explain differences in SPT rates and to understand how SPT can be prevented, while maintaining a high intact perineum rate.

## 1 Introduction

Perineal injury is common during vaginal birth and can be categorized into first-, second-, third- and fourth-degree tears. First-degree tears only involve the perineal skin, while second-degree tears also involve the perineal muscles. A perineal injury involving the anal sphincter is defined as a third- or fourth-degree tear, with the latter extending to the rectal mucosa. Both third- and fourth-degree tears are defined as severe perineal trauma (SPT), also known as obstetric anal sphincter injury (OASI) [1,2]. SPT resulting from vaginal birth is a severe outcome for women with short- and long-term consequences. Women with SPT report pelvic floor pain and sexual dissatisfaction more frequently three months after birth, compared to women with second-degree perineal injury [3]. Furthermore, among women with SPT, a higher incidence of fecal incontinence later in life has been reported [4], which is likely to have a substantial negative impact on quality of life [5].

The incidence of SPT has increased in many countries over the last decades [6]. Known risk factors for SPT are primiparity, assisted vaginal birth (e.g. birth with vacuum or forceps), prolonged duration of the second stage of labour, occiput-posterior position, previous CS, and higher birthweight [[7], [8], [9], [10]]. Additionally, more attention for the appropriate diagnosis of SPT [11] may have resulted in increased detection and thereby seemingly rising rates in some countries [12]. Also, a decreasing episiotomy rate may be associated with an increase in SPT [13]. In other countries the incidence of SPT appears to have decreased, perhaps due to increased awareness of its possible impact and options for prevention [14,15]. The registered incidence of SPT is therefore not only influenced by maternal, neonatal, and birth-related characteristics, but also by labour management and the skills of health care providers in diagnosing it.

The incidence of SPT not only differs between countries, but may also differ between maternity care settings within a country. In a systematic review, no differences were found in rates of perineal tears resulting in surgical repair between midwife-led continuity models of care, compared to other models of care, but differences in SPT-rates were not described [16]. Conflicting results have been described with regard to the incidence of SPT among women in midwife-led care planning birth at home compared to those planning birth in a hospital [17,18]. Differences in SPT rates between maternity care settings may for instance be due to differences in maternal and neonatal characteristics, the course of labour and birth, birth interventions, care providers’ awareness of the diagnosis, or management in the second stage of labour. There are indications that the incidence of SPT in the Netherlands is higher in midwife-led care compared to obstetrician-led care, which is not expected when considering the higher risk profile of women giving birth in obstetrician-led care [19]. It is important to gain insight into the factors that are associated with SPT in each setting as this may explain the difference in incidence. We therefore aimed to investigate what the exact differences in SPT incidence between midwife-led and obstetrician-led care are stratified for nulliparous and multiparous women between 2010 and 2019, and how the trend on SPT in both care settings evolved over time in the period 2000–2019. Secondly, we aimed to examine which factors are associated with SPT in both care settings.

## 2 Methods

### 2.1 Data collection

This nationwide cohort study was conducted in the Netherlands with data from the national perinatal registry Perined. In this database, data for 98 % of births in the Netherlands are collected into three sub-registers that are linked via a validated linkage method [20,21]. These three sub registers contain data from births registered by care providers in midwife-led care (LVR 1), obstetrician-led care (LVR 2), and paediatric care (LNR). In advance of data collection, women gave written consent for usage of the data in the perinatal registry. In this study, data were fully anonymized before accessed by the researchers.

### 2.2 Inclusion criteria and setting

We included spontaneous vaginal births of a term (37–42 weeks), live, cephalic, single infant between 2000 and 2019 registered in the perinatal database. Women with a CS, previous CS, or assisted vaginal birth were excluded from all analyses, since these do not occur in midwife-led care and largely impact the risk of SPT. Cases with missing information on presence of perineal injury, mode of birth, parity, or maternity care setting were excluded.

In the Netherlands, women with low-risk pregnancies generally receive midwife-led care, which means that autonomously working midwives in the community attend women during birth; this is also referred to as primary care [22]. Pregnancy and birth are regarded as low risk as long as no referral to obstetrician-led care is required. Criteria for referral to obstetrician-led care are described in the Dutch obstetric indication list of 2003 [23]. Women in midwife-led care can choose to give birth at home or in hospital. When the chance of complications is increased or complications occur, women are transferred to obstetrician-led care, also described as secondary or tertiary care. In obstetrician-led care, birth is attended by hospital-based midwives, obstetric registrars, and/or obstetricians. Interventions such as induction or augmentation of labour with oxytocin, assisted vaginal birth, pharmacological pain medication, and caesarean section (CS) are only provided in obstetrician-led care. Interventions such as episiotomy, artificial rupture of membranes, and suturing of episiotomy and perineal injuries are applied in both midwife-led and obstetrician-led care, whereas suturing of SPT is only performed in obstetrician-led care.

### 2.3 Selection of variables

Combined variables based on the three sub-registers are created by Perined. Some combined variables were not appropriate for our study. We therefore redefined the variables ethnic background, socioeconomic status, augmentation of labour, and pain medication. The redefined definitions are described below.

#### 2.3.1 Dependent variable

The main outcome of interest was SPT, defined as a third- or fourth degree perineal tear diagnosed by care providers.

#### 2.3.2 Independent variables

The following population characteristics were described: parity (primiparous; multiparous), maternal age (<25; 25–29; 30–34; 35–39; ≥40 years), ethnic background (Western; non-Western), socioeconomic status (low; middle; high); gestational age (37+0–37 + 6; 38+0–40 + 6; 41+0–41 + 6 weeks); birthweight (<3000; 3000–3999; ≥4000 g); duration of the second stage (<30; 30–59; ≥60 min); shoulder dystocia (yes; no); place of birth (home; hospital), induction of labour (yes; no), augmentation after spontaneous onset of labour (yes; no), pain medication (none; epidural; other), episiotomy (yes; no), and intact perineum (yes; no).

Ethnic background was based on the registration by the care provider. ‘Western ethnic background’ comprised a Dutch, white European, or other ‘Western’ ethnic background, such as North-American or Australian. Non-Western was defined as all other registered ethnic backgrounds.

If birth took place in a birth center, this was categorized as ‘in hospital’ in the variable ‘place of birth’.

Induction of labour was defined as all pharmacological and mechanical methods to stimulate uterine contractions prior to spontaneous rupture of membranes or uterine contractions. Augmentation of labour was defined as the use of oxytocin during labour, after a spontaneous onset of labour. Other pharmacological methods for pain relief comprise sedatives, non-opioid analgesia, or opioid analgesia. In practice, pethidine injections or patient-controlled remifentanil are the most frequently used methods other than epidural analgesia [24]. In the Netherlands, most episiotomies (approximately 99 %) are mediolateral. Intact perineum was defined as the absence of a perineal or vaginal tear but women may have had labial tears.

Not all independent variables were registered in both the LVR 1 and LVR 2 registers or applicable to both maternity care settings. This was the case for shoulder dystocia (only registered in LVR 2). Therefore, adjustment for shoulder dystocia was not applied in the comparison between the health care settings. Place of birth is only applicable to midwife-led care, since women in obstetrician-led care always gave birth in the hospital. The interventions induction of labour, augmentation of labour, and pain medication are only applicable to obstetrician-led care.

### 2.4 Data analyses

First, we showed the trend in the incidence of SPT and that of episiotomy from 2000 to 2019 in midwife-led and obstetrician-led care. We also showed the trend in the incidence of intrapartum CS and assisted vaginal birth for women who were in midwife-led versus obstetrician-led care at the onset of labour, because the change in these intervention rates will have influenced the included population over time. Second, we identified differences in the rate of SPT between midwife-led and obstetrician-led care in the period 2010 to 2019. We chose a shorter time period, because in this period an relatively stable difference in the rate of SPT between midwife-led and obstetrician-led care was seen, and analyses within this time period will give insight in more recent numbers. Third, we investigated which factors in obstetrician-led care could be associated with the differences in incidences of SPT between the care settings. Because these factors were not available in midwife-led care, we could not adjust for them in the primary analyses, and therefore, we analyzed these factors separately for women in obstetrician-led care only. We also examined the association between place of birth and SPT in midwife-led care only, in order to gain more insight into the role of place of birth in the occurrence of SPT.

Characteristics of the study population were described in numbers and percentages, stratified for midwife-led and obstetrician-led care. Descriptive analyses were also used to calculate the trend incidences of SPT and we tested the significance of the trends with the Chi-square test. All calculated associations with the outcome measure were stratified for primiparous and multiparous women, because parity modifies the associations between the health care setting and the SPT rate. Uni- and multivariable logistic regression analyses were performed to calculate the crude and adjusted odds ratios (OR) with 99 % confidence intervals (CI) for the association between the maternity care setting (independent variable) and SPT (dependent variable). A 99 % CI was chosen to account for multiple testing in a large data set. ORs for the outcome SPT were calculated for births in obstetrician-led care, compared to midwife-led care (reference category). For the association between the maternity care setting and SPT, and for the association between place of birth and SPT, adjustments were made in two steps: first for maternal age, ethnic background, socioeconomic status, gestational age, birthweight, and duration of the second stage, and second, for episiotomy as well. Potential confounders were chosen based on the existing evidence from the literature [[25], [26], [27]]. Subgroup analyses were performed to estimate the association between the maternity care setting and SPT among women without epidural analgesia and among women without shoulder dystocia, induction or augmentation of labour, and pain medication.

Uni- and multivariable logistic regression analyses were performed to calculate the crude and adjusted OR with 99 % CIs for the association between care factors (independent variable) and SPT (dependent variable) in obstetrician-led care. For these associations, a third adjustment step was added. Adjustments were made in the following sequence: first for the aforementioned characteristics and shoulder dystocia; second, birth interventions were added to the model, and third, the analyses were also adjusted for episiotomy. We performed these separate adjustment steps in order to gain insight into which factors have the strongest association with SPT and may be a factor that explains the differences in SPT incidence between midwife-led and obstetrician-led care.

Because the continuous confounder variables maternal age, gestational age, birthweight, and duration of the second stage were not linearly associated with the log odds of SPT, we used restricted cubic splines with five knots in the multivariable analyses [28].

The percentage of missing data was lower than 5 % for all variables and therefore, multiple imputation was not performed. Statistical analyses were performed using STATA version 14 (StataCorp, Texas, USA).

## 3 Results

### 3.1 Study population and trend

**Fig. 1:**
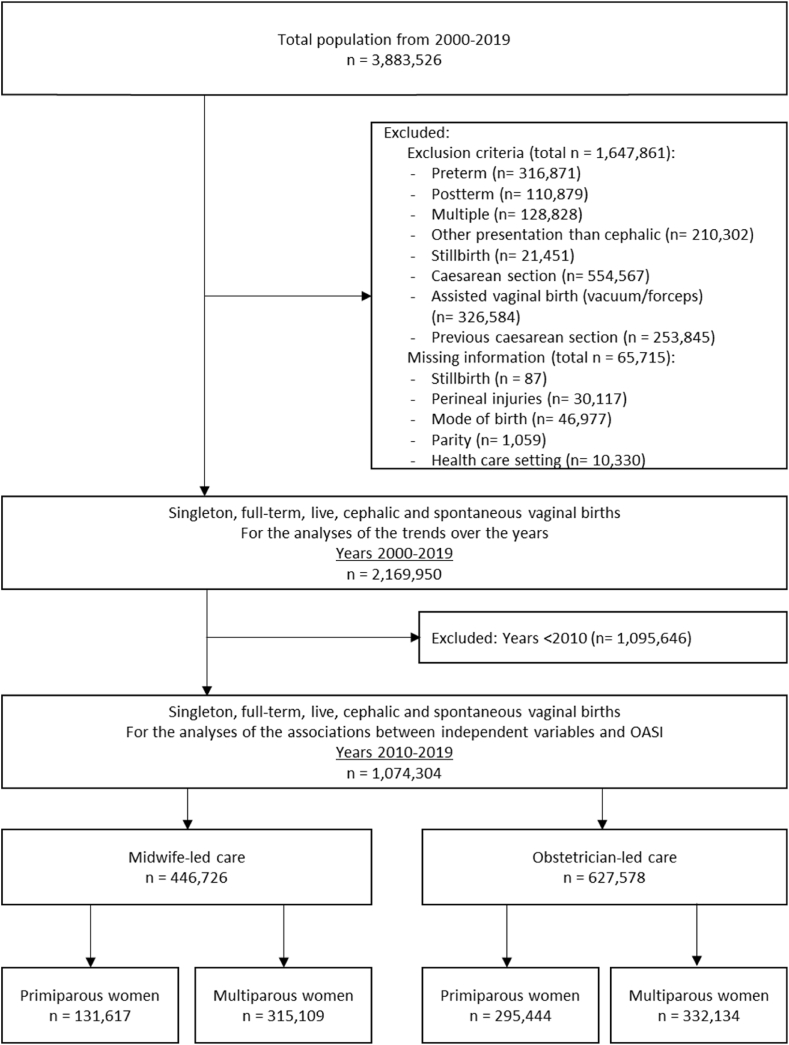
Flowchart of the study population.

**Fig. 2:**
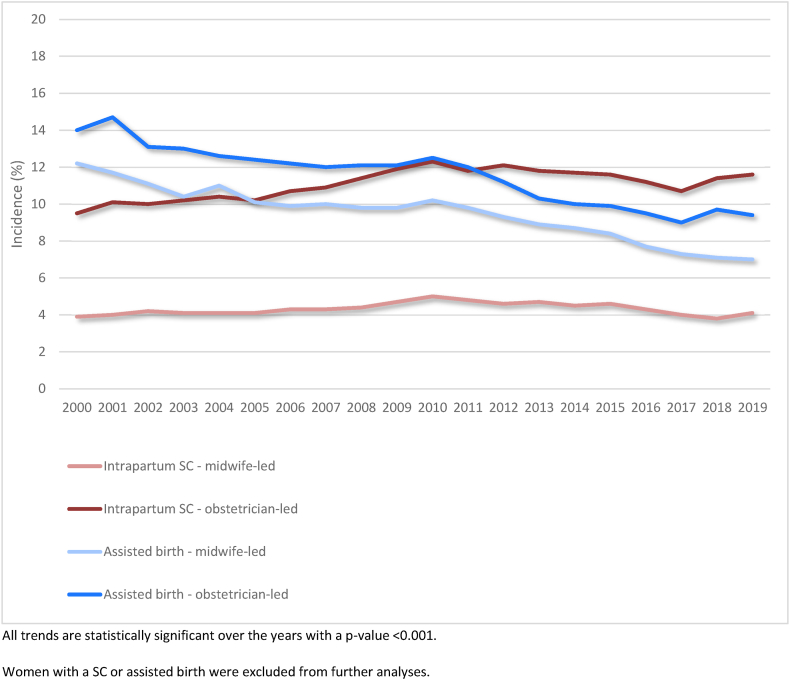
Trend in intrapartum caesarean section and assisted vaginal birth in midwife-led and obstetrician-led care at the onset of labour over the years 2000–2019

This study contained 2,169,950 births in the period 2000 to 2019 (Fig. 1). The incidence of assisted vaginal births and intrapartum CS are shown in Fig. 2. In further analyses, women with an assisted vaginal birth or CS were excluded. This figure shows a significant decrease in assisted vaginal births and increase in intrapartum caesarean sections, with higher incidences of both interventions in obstetrician-led care, compared to midwife-led care at the onset of labour.

**Fig. 3:**
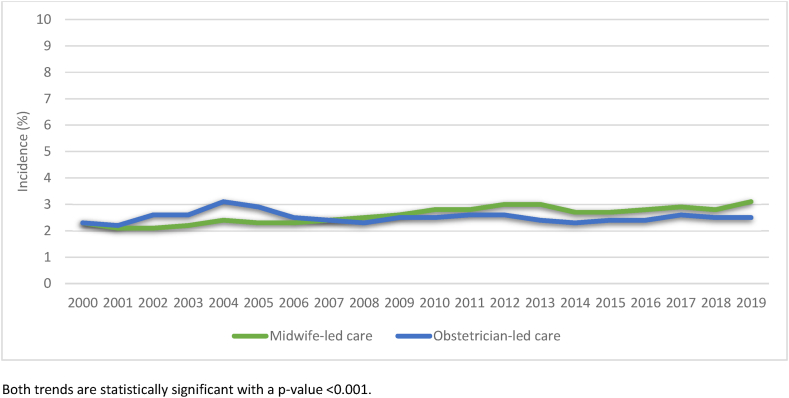
Trend in SPT for births in midwife-led and obstetrician-led care over the years 2000–2019

**Fig. 4:**
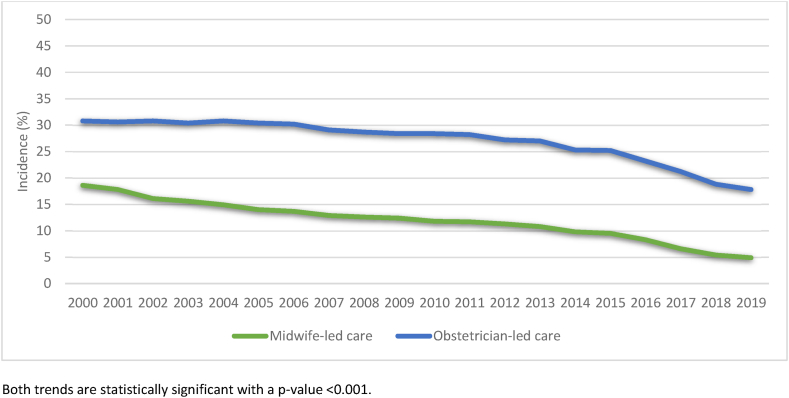
Trend in episiotomy for births in midwife-led and obstetrician-led care over the years 2000–2019

From 2000 to 2006, the incidence of SPT was lower in midwife-led care compared to obstetrician-led care (mean of 2.2 % versus 2.6 %). However, from 2008 onwards, the incidence was higher in midwife-led care compared to obstetrician-led care (2.8 % versus 2.5 %) (Fig. 3). In both care settings, the incidence of episiotomy decreased: from 18.6 % in 2000 to 4.9 % in 2019 in midwife-led care, and from 30.8 % to 17.8 % in obstetrician-led care (<p 0.001; Fig. 4). In the period 2010 to 2019, a total of 1,074,304 births were included, of which 446,726 were in midwife-led care (41.6 %) and 627,578 in obstetrician-led care (58.4 %) (Fig. 1). SPT occurred in 2.6 % of all births (Table 2), episiotomies were performed in 18.0 % (Table 1).

**Table 1:** Characteristics of women in midwife- and obstetrician-led care at the time of birth, 2010–2019.

Total		*Total n (%)*	Midwife-led care n (%)	Obstetrician-led care n (%)
		1,074,304	446,726 (41.6)	627,578 (58.4)
Parity	Primiparous	427,061 (39.8)	131,617 (29.5)	295,444 (47.1)
	Multiparous	647,243 (60.2)	315,109 (70.5)	332,134 (52.9)
Maternal age	<25 years	116,905 (10.9)	37,407 (8.4)	79,498 (12.7)
	25–29 years	334,003 (31.1)	136,621 (30.6)	197,382 (31.4)
	30–34 years	411,202 (38.3)	186,119 (41.7)	225,083 (35.9)
	35–39 years	181,401 (16.9)	76,512 (17.1)	104,889 (16.7)
	≥40 years	30,753 (2.8)	10,050 (2.2)	20,703 (3.3)
Ethnic background	Western	971,518 (91.2)	402,396 (91.6)	569,122 (91.0)
	Non-Western	93,514 (8.8)	37,102 (8.4)	56,412 (9.0)
Socioeconomic status	Low	283,678 (26.7)	105,132 (24.0)	178,546 (28.7)
	Middle	505,953 (47.7)	215,636 (49.1)	290,317 (46.7)
	High	271,521 (25.6)	118,284 (26.9)	153,237 (24.6)
Gestational age	37 + 0–37 + 6 weeks	75,840 (7.1)	17,716 (4.0)	58,124 (9.3)
	38 + 0–40 + 6 weeks	803,509 (74.8)	355,117 (79.5)	448,392 (71.4)
	41 + 0–41 + 6 weeks	194,955 (18.1)	73,893 (16.5)	121,062 (19.3)
Birthweight	<3000 g	145,715 (13.6)	42,262 (9.5)	103,453 (16.5)
	3000–3999 g	774,862 (72.2)	334,541 (75.1)	440,321 (70.2)
	≥4000 g	152,448 (14.2)	68,886 (15.4)	83,562 (13.3)
Duration second stage of labour	<30 min	735,621 (70.3)	323,554 (74.8)	412,067 (67.2)
	30–59 min	190,735 (18.2)	66,867 (15.4)	123,868 (20.2)
	≥60 min	119,829 (11.5)	42,390 (9.8)	77,439 (12.6)
Shoulder dystocia	Yes	–	–	11,265 (1.8)
	No	–	–	616,313 (98.2)
Place of birth	Home	–	226,681 (51.0)	–
	Hospital	–	218,108 (49.0)	627,578 (100.0)
Induction of labour	Yes	–	–	225,321 (36.4)
	No	–	–	393,900 (63.6)
Augmentation after spontaneous onset of labour	Yes	–	–	202,029 (51.2)
	No	–	–	192,962 (48.9)
Pain medication^a^	None	–	–	297,086 (48.3)
	Epidural	–	–	169,049 (27.5)
	Other	–	–	167,042 (27.2)
Episiotomy	Yes	192,944 (18.0)	40,381 (9.0)	152,563 (24.3)
	No	881,360 (82.0)	406,345 (91.0)	475,015 (75.7)
Intact perineum	Yes	376,510 (35.0)	170,985 (38.3)	205,525 (32.8)
	No	697,794 (65.0)	275,741 (61.7)	422,052 (67.3)

Percentages of missing data: 0.9 % for ethnic background, 1.2 % for socio-economic status, 0.1 % for birthweight, 2.6 % for duration second stage of labour, 0.8 % for induction or augmentation of labour, 1.1 % for pain medication; all other: 0 %.

a Pain medication during labour: total numbers exceed the total number of births in the study, because more than one method can be used.

**Table 2:** Uni- and multivariable analyses of Severe Perineal Trauma in obstetrician-led compared to midwife-led care at the time of birth.

Total	Total		Primiparous women n = 427,061				Multiparous women n = 647,243			
	Total N	n of SPT (%)	n (%)	OR (99 % CI)	aOR^a^ (99 % CI)	aOR^b^ (99 % CI)	n (%)	OR (99 % CI)	aOR^a^ (99 % CI)	aOR^b^ (99 % CI)
	1,074,304	28,089 (2.6)	17,914 (4.2)				10,175 (1.6)			
Midwife-led care	**446,726**	12,699 (2.8)	7476 (5.7)	Ref	Ref	Ref	5223 (1.7)	Ref	Ref	Ref
**Obstetrician-led care**										
All women	**627,578**	15,390 (2.5)	10,438 (3.5)	**0.61 (0.58**–**0.63)**	**0.62 (0.60**–**0.65)**	**0.78 (0.74**–**0.81)**	4952 (1.5)	**0.90 (0.85**–**0.95)**	0.96 (0.91–1.02)	1.04 (0.99–1.10)
Women without epidural analgesia	**446,336**	11,026 (2.5)	6866 (3.9)	**0.68 (0.65**–**0.71)**	**0.70 (0.67**–**0.74)**	**0.88 (0.84**–**0.92)**	4160 (1.5)	**0.92 (0.87**–**0.97)**	1.01 (0.95–1.07)	**1.09 (1.03**–**1.15)**
Women without shoulder dystocia, induction, augmentation, pain medication	**124,664**	3162 (2.5)	1740 (4.1)	**0.70 (0.65**–**0.75)**	**0.75 (0.70**–**0.81)**	0.93 (0.86–1.001)	1422 (1.7)	1.05 (0.97–1.14)	**1.19 (1.10**–**1.29)**	**1.26 (1.16**–**1.37)**

a Adjusted for maternal age, ethnic background, socioeconomic status, gestational age, birthweight, and duration second stage.

b Additionally adjusted for episiotomy.

## 4 Characteristics and associations with SPT

Compared to births in midwife-led care, births in obstetrician-led care were more often of primiparous women, women with a low socioeconomic status, and of infants with a birthweight below 3000 g (Table 1). Births of women aged 30–34 years, between 38 + 0 and 40 + 6 weeks of gestation, and with a shorter duration of the second stage of labour were more common in midwife-led compared to obstetrician-led care. Of the births in midwife-led care 51.0 % took place at home.

Episiotomies were performed in 9.0 % of births in midwife-led care, compared to 24.3 % in obstetrician-led care. Women in midwife-led care more often had an intact perineum (38.3 %), compared to women in obstetrician-led care (32.8 %).

## 5 Maternity care settings

The SPT incidence was lower in obstetrician-led care compared to midwife-led care among primiparous women (3.5 versus 5.7 %) and multiparous women (1.5 versus 1.7 %) (Table 2). After adjusting for characteristics, there was a significantly lower odds of STP in obstetrician-led compared to midwife-led care among primiparous women, but no statistically significant difference among multiparous women. After further adjusting for episiotomy there was a decreased odds of STP in obstetrician-led care among primiparous women (0.78; 99 % CI 0.74–0.81), and no difference among multiparous women (1.04; 99 % CI 0.99–1.10). Adjusting for episiotomy resulted in higher odds ratios, nearer to ‘no difference’, compared to the odds ratios before adjustments.

In the subgroup analyses, comparisons in SPT rate between midwife-led and obstetrician-led care were made for births without the use of epidural analgesia, and for births without shoulder dystocia, induction, augmentation, or pain medication. Differences in SPT rate between midwife- and obstetrician-led care were smaller in these subgroup analyses (Table 2). After adjusting for characteristics and for episiotomy, there were no differences among primiparous women without shoulder dystocia, induction, augmentation, or pain medication in obstetrician-led care, in comparison to midwife-led care. Multiparous women in obstetrician-led care in these subgroups had higher rates of SPT, compared to those in midwife-led care.

### 5.1 Midwife-led care

Within the midwife-led setting, SPT occurred more often in hospital births, compared to home births in both primiparous (6.2 % versus 5.1 %), and multiparous women (2.0 % versus 1.3 %) (data not shown in Tables). Also, after adjustments for population characteristics and episiotomy, giving birth in a hospital was associated with a higher incidence of SPT among both primiparous (aOR 1.29; 99 % CI 1.23–1.38) and multiparous women (aOR 1.57; 99 % CI 1.46–1.70) compared to giving birth at home.

### 5.2 Obstetrician-led care

Table 3 shows the associations of care factors in obstetrician-led care with SPT. These care factors may be related to the difference in SPT rate between midwife-led and obstetrician-led care.

**Table 3:** Uni- and multivariable analyses of factors associated with SPT only applicable in obstetrician-led care or available in LVR2.

		Obstetrician-led care n = 295,444				
		n (%)	OR (99 % CI)	aOR^a^ (99 % CI)	aOR^b^ (99 % CI)	aOR^c^(99 % CI)
***Primiparous women***						
Shoulder dystocia	Yes	233 (7.3)	**2.16 (1.81**–**2.58)**	**1.40 (1.17**–**1.68)**	**1.40 (1.16**–**1.68)**	**1.56 (1.30**–**1.89)**
	No	10,205 (3.5)	Ref	Ref	Ref	Ref
Induction of labour	Yes	2711 (3.2)	**0.86 (0.81**–**0.91)**	**0.93 (0.88**–**0.99)**	0.97 (0.91–1.03)	0.97 (0.91–1.03)
	No	7606 (3.7)	Ref	Ref	Ref	Ref
Augmentation after spontaneous onset of labour	Yes	4689 (3.6)	0.97 (0.91–1.03)	**0.88 (0.82**–**0.93)**	0.98 (0.92–1.05)	0.99 (0.93–1.06)
	No	2893 (3.7)	Ref	Ref	Ref	Ref
Pain medication^d^	None	4233 (4.1)	Ref	Ref	Ref	Ref
	Epidural	3354 (2.9)	**0.73 (0.69**–**0.77)**	**0.72 (0.68**–**0.76)**	**0.70 (0.66**–**0.74)**	**0.68 (0.63**–**0.72)**
	Other	3015 (3.6)	1.04 (0.99–1.11)	1.02 (0.97–1.09)	**0.91 (0.85**–**0.97)**	**0.90 (0.85**–**0.96)**
***Multiparous women***						
Shoulder dystocia	Yes	322 (4.0)	**2.87 (2.47**–**3.34)**	**1.53 (1.30**–**1.80)**	**1.43 (1.16**–**1.76)**	**1.56 (1.32**–**1.84)**
	No	4630 (1.4)	Ref	Ref	Ref	Ref
Induction of labour	Yes	1730 (1.2)	**0.73 (0.68**–**0.79)**	**0.80 (0.73**–**0.87)**	**0.82 (0.75**–**0.88)**	**0.82 (0.75**–**0.90)**
	No	3151 (1.7)	Ref	Ref	Ref	Ref
Augmentation of labour	Yes	1153 (1.6)	0.92 (0.83–1.01)	**0.81 (0.74**–**0.90)**	**0.85 (0.77**–**0.94)**	**0.86 (0.78**–**0.95)**
	No	2008 (1.7)	Ref	Ref	Ref	Ref
Pain medication^d^	None	3020 (1.6)	Ref	Ref	Ref	Ref
	Epidural	699 (1.3)	**0.85 (0.76**–**0.95)**	**0.76 (0.68**–**0.85)**	**0.79 (0.70**–**0.88)**	**0.78 (0.70**–**0.88)**
	Other	1198 (1.4)	0.94 (0.86–1.02)	0.92 (0.85–1.01)	0.92 (0.84–1.00)	0.93 (0.84–1.01)

a Adjusted for maternal and birth characteristics (maternal age, ethnic background, socioeconomic status, gestational age, birthweight, duration second stage of labour, and shoulder dystocia).

b Adjusted for maternal and birth characteristics and additionally adjusted for birth interventions except episiotomy (induction or augmentation of labour, and pain medication).

c Adjusted for maternal and birth characteristics, birth interventions, and additionally adjusted for episiotomy.

d Pain medication during labour: total numbers exceeds the total number of births in the study, because more than one method can be used. Reference for epidural is ‘no epidural’; reference for other pain medication is ‘no other pain medication’.

SPT occurred more often among births complicated by shoulder dystocia (7.3 % versus 3.5 % among primiparous and 4.0 % versus 1.4 % among multiparous women), but this association became less pronounced after adjusting for confounding factors (aOR 1.56; 99 % CI 1.30–1.89 among primiparous and 1.56; 99 % CI 1.32–1.84 among multiparous women). Although among primiparous women a statistically significant lower OR of SPT was shown for induced births, this OR was no longer significant after adjustment (aOR 0.97; 99 % CI 0.91–1.03). Among multiparous women, induced birth was associated with a statistically significant lower incidence of SPT (1.2 % versus 1.7 %), also after adjusting (aOR 0.82; 99 % CI 0.75–0.90). For augmented births, the SPT rate was similar (1.6 % versus 1.7 %), but became significantly lower after adjustment (aOR 0.86; 99 % CI 0.78–0.95).

Among both primiparous and multiparous women, SPT occurred less often in births with the use of epidural analgesia (2.9 % versus 4.1 %, aOR 0.68; 99 % CI 0.63–0.72 among primiparous women and 1.3 % versus 1.6 %, aOR 0.78; 99 % CI 0.70–0.88 among multiparous women). The use of other pharmacological methods for pain relief was associated with a significantly lower incidence of SPT among primiparous (3.6 % versus 4.1 %, aOR 0.90; 99 % CI 0.85–0.96), but not among multiparous women (aOR 0.93, 99 % CI 0.84–1.01).

Overall, adjusting for episiotomy did not lead to large changes in the ORs in the associations of all care factors with SPT.

## 6 Discussion

### 6.1 Main findings

This nationwide cohort study with data of spontaneous vaginal births in the Netherlands showed a rising trend in incidence of SPT in midwife-led care and a decreasing trend in episiotomy rate between 2000 and 2019. Compared to midwife-led care, giving birth in obstetrician-led care was associated with a lower incidence of SPT among primiparous women and a comparable incidence among multiparous women. However, among births without shoulder dystocia, induction, augmentation, or pain medication, the incidence of SPT in obstetrician-led care was comparable to midwife-led care among primiparous women, and higher among multiparous women. Higher incidences of SPT were found for hospital versus home birth in midwife-led care, and for shoulder dystocia in obstetrician-led care. Lower incidences were found for induced and augmented births among multiparous women, and epidural analgesia among both primiparous and multiparous women.

### 6.2 Strengths and limitations

This was the first study showing trends of SPT over the last two decades in the Netherlands in both midwife-led and obstetrician-led care. A strength is the inclusion of almost all births in the Netherlands in the included time period, enabling subgroup analyses and analyses of primiparous and multiparous women separately, and enabling multivariable analyses to investigate associations with a rare outcome.

A disadvantage of making use of a registration-based dataset was that we could not adjust for all possible confounders, such as body mass index, specific ethnic groups, occiput posterior position, birthing positions, and the experience of the care provider [25,26,29,30]. Also, not all previous CS had been registered and therefore, some multiparous women with a previous CS will still have been included in the dataset. However, we could exclude most women with a previous CS and could adjust for other important characteristics. We showed that these characteristics did not explain the differences in SPT rate between the care settings. Furthermore, 2 % of women giving birth in the included period in the Netherlands, were not included in the registry. Further research is needed into the associations of birthing positions and the level of experience of the care provider, and the occurrence of SPT.

### 6.3 Interpretation

Our findings are consistent with results from Ireland where a higher SPT rate was found among primiparous women giving birth in midwife-led units [31,32], compared to obstetrician-led units, but they showed a lower incidence among multiparous women [31]. However, other studies did not show a difference in SPT rates between health settings [33] or showed lower rates in midwife-led care regardless of parity [34]. A systematic review confirmed our results of higher SPT rates among hospital births compared to home births in midwife-led care settings [17].

More often than in midwife-led care, births in obstetrician-led care are influenced by risk factors such as higher birthweight, prolonged second stage of labour, occiput posterior position, and shoulder dystocia, which were found to be associated with a higher chance of having SPT in our study and the literature [25,29]. Therefore, a higher incidence of SPT would be expected in obstetrician-led care. It is therefore remarkable that among primiparous women SPT occurred less often in obstetrician-led care compared to midwife-led care, even after adjusting for characteristics. There are several possible explanations for this finding.

First, we excluded women with CS or assisted vaginal birth, since these do not occur in midwife-led care. Therefore, women in our study in obstetrician-led care may have a lower risk profile for SPT, compared to the general population in obstetrician-led care. Moreover, CS rates are higher for women in obstetrician-led care at the onset of labour. We showed a rising trend in intrapartum CS in obstetrician-led care and a decreasing trend in assisted births.

Second, the use of epidural analgesia, which is only administered in obstetrician-led care, was associated with a lower SPT incidence, which is consistent with previous studies [[35], [36], [37]]. The incidence of SPT in subgroup analyses of births without epidural analgesia in our study were indeed higher compared to the group including births with epidural analgesia. Women with epidural analgesia may have an increased relaxation of the perineal muscles and a slower passage of the fetal head through the vulva due to a reduced urge to push. These factors may decrease the risk of SPT [38]. Further research is needed to give more insight into the mechanisms of the possible preventive effect of epidural analgesia on SPT.

Third, episiotomies are more common in obstetrician-led care and may be associated with a lower incidence of SPT. However, the higher episiotomy rate did not entirely explain the lower SPT incidence in obstetrician-led care, because after adjustments for episiotomy, the difference among primiparous women between the care settings remained. High episiotomy rates are not recommended [1,39] because of disadvantages such as pain, discomfort, sexual problems [40], and a lower chance of intact perineum, which is an important outcome for women as well [41]. Other non-harmful methods to prevent SPT are recommended, such as the use of warm compresses during the second stage of labour, supporting a slow passage of the fetal head, and the assistance of a second midwife during the second stage of labour [38,[42], [43], [44]].

Place of birth may be another factor explaining a higher SPT rate in midwife-led care among primiparous women, because home births are only possible in midwife-led care. However, we showed that giving birth in the hospital was associated with a higher incidence of SPT compared to home birth. Previous literature showed conflicting results on place of birth and the occurrence of SPT [17,18].

Other possible explanations for the differences in SPT rates between the care settings may be a difference in perineal management and differences in the use of different birthing positions. However, further research is needed to gain a deeper understanding in differences in perineal management and birthing positions and their impact on SPT in the different maternity care settings.

## 7 Conclusions

This nationwide cohort study showed differences in the rate of SPT between midwife-led and obstetrician-led care, and showed possible explanations for these differences. It provides valuable leads for among spontaneous vaginal births improving care related to perineal trauma. In midwife-led care, the incidence of SPT has increased in the last decades. Compared to obstetrician-led care, the incidence of SPT in midwife-led care is higher among primiparous women, and similar among multiparous women. However, among women without shoulder dystocia, induction, augmentation, or pain medication, there was no difference in SPT rate among primiparous women, and among multiparous women, the incidence was lower in midwife-led care. Concurrently, a higher incidence of episiotomy and lower rate of intact perineum was found among primiparous and multiparous women in obstetrician-led care, but episiotomy did not fully explain the differences in SPT. Higher incidences of SPT were found for hospital births compared to home births, and births complicated by shoulder dystocia. Lower incidences of SPT were found in induced and augmented births among multiparous women, and in births with epidural analgesia in both primiparous and multiparous women. Interventions only used in obstetrician-led care (induction, augmentation, and epidural analgesia) may be an explanatory factor for the higher incidence of SPT among primiparous women in midwife-led care. More research is needed to explain these differences in SPT rate in the different care settings to understand how SPT can be prevented, while maintaining a high intact perineum rate.

## Data availability

The authors do not have permission to share data.

The data that support the findings of this study are available from Perined (contact via info@perined.nl) for researchers who meet the criteria for access to the confidential data, and if Perined gives permission. Restrictions apply to the availability of these data, which were used under license for this study.

## Ethics declarations

This study was reviewed and approved by the ethics committee of The VU University Medical Center, with the approval number: 2020.181.

All participants provided informed consent for routinely collecting data in the Perined-database, and thereby provided informed consent for the use of their data in this study.

## CRediT authorship contribution statement

**Anna E. Seijmonsbergen-Schermers:** Writing – original draft, Visualization, Validation, Project administration, Methodology, Investigation, Formal analysis, Conceptualization. **Kelly MCM. Peerdeman:** Project administration, Methodology, Investigation, Formal analysis, Conceptualization. **Thomas van den Akker:** Writing – review & editing, Conceptualization. **Linde ML. Titulaer:** Writing – review & editing. **Jan-Paul Roovers:** Writing – review & editing. **Lilian L. Peters:** Writing – review & editing, Supervision, Formal analysis, Conceptualization. **Corine J. Verhoeven:** Writing – review & editing, Supervision, Conceptualization. **Ank de Jonge:** Writing – review & editing, Supervision, Conceptualization.

## Declaration of competing interest

The authors declare that they have no known competing financial interests or personal relationships that could have appeared to influence the work reported in this paper.

## References

[1] Steen M., Diaz M. (2018). Perineal trauma: a women's health and wellbeing issue. Br. J. Midwifery.

[2] Royal College of Obstetricians & Gynaecologists (2015). https://www.rcog.org.uk/media/5jeb5hzu/gtg-29.pdf.

[3] Klein M.C., Gauthier R.J., Robbins J.M., Kaczorowski J., Jorgensen S.H., Franco E.D. (1994). Relationship of episiotomy to perineal trauma and morbidity, sexual dysfunction, and pelvic floor relaxation. Am. J. Obstet. Gynecol..

[4] Bols E.M., Hendriks E.J., Berghmans B.C., Baeten C.G., Nijhuis J.G., de Bie R.A. (2010). A systematic review of etiological factors for postpartum fecal incontinence. Acta Obstet. Gynecol. Scand..

[5] Cornelisse S., Arendsen L.P., van Kuijk S.M., Kluivers K.B., van Dillen J., Weemhoff M. (2016). Obstetric anal sphincter injury: a follow-up questionnaire study on longer-term outcomes. Int. Urogynecol. J..

[6] Blondel B., Alexander S., Bjarnadottir R.I., Gissler M., Langhoff-Roos J., Novak-Antolic Z. (2016). Euro-Peristat Scientific Committee. Variations in rates of severe perineal tears and episiotomies in 20 European countries: a study based on routine national data in Euro-Peristat Project. Acta Obstet. Gynecol. Scand..

[7] Dudding T.C., Vaizey C.J., Kamm M.A. (2008). Obstetric anal sphincter injury: incidence, risk factors, and management. Ann. Surg..

[8] Smith L.A., Price N., Simonite V., Burns E.E. (2013). Incidence of and risk factors for perineal trauma: a prospective observational study. BMC Pregnancy Childbirth.

[9] Meister M.R., Cahill A.G., Conner S.N., Woolfolk C.L., Lowder J.L. (2016). Predicting obstetric anal sphincter injuries in a modern obstetric population. Am. J. Obstet. Gynecol..

[10] Marschalek M.L., Worda C., Kuessel L., Koelbl H., Oberaigner W., Leitner H. (2018). Risk and protective factors for obstetric anal sphincter injuries: a retrospective nationwide study. Birth.

[11] Ginath S., Mizrachi Y., Bar J., Condrea A., Kovo M. (2017). Obstetric anal sphincter injuries (OASIs) in Israel: a review of the incidence and risk factors. Rambam Maimonides Med. J..

[12] Sioutis D., Thakar R., Sultan A.H. (2017). Overdiagnosis and rising rate of obstetric anal sphincter injuries (OASIS): time for reappraisal. Ultrasound Obstet. Gynecol..

[13] Ghulmiyyah L., Sinno S., Mirza F., Finianos E., Nassar A.H. (2022). Episiotomy: history, present and future - a review. J. Matern. Fetal Neonatal Med..

[14] Gyhagen M., Ellstrom Engh M., Husslein H., Koelbl H., Nilsson I.E.K., Schulz J. (2021). Temporal trends in obstetric anal sphincter injury from the first vaginal delivery in Austria, Canada, Norway, and Sweden. Acta Obstet. Gynecol. Scand..

[15] Gurol-Urganci I., Bidwell P., Sevdalis N., Silverton L., Novis V., Freeman R. (2021). Impact of a quality improvement project to reduce the rate of obstetric anal sphincter injury: a multicentre study with a stepped-wedge design. Bjog.

[16] Sandall J., Soltani H., Gates S., Shennan A., Devane D. (2016). Midwife-led continuity models versus other models of care for childbearing women. Cochrane Database Syst. Rev..

[17] Scarf V.L., Rossiter C., Vedam S., Dahlen H.G., Ellwood D., Forster D. (2018). Maternal and perinatal outcomes by planned place of birth among women with low-risk pregnancies in high-income countries: a systematic review and meta-analysis. Midwifery.

[18] Bolten N., de Jonge A., Zwagerman E., Zwagerman P., Klomp T., Zwart J.J. (2016). Effect of planned place of birth on obstetric interventions and maternal outcomes among low-risk women: a cohort study in The Netherlands. BMC Pregnancy Childbirth.

[19] Perined (2021). https://www.peristat.nl/.

[20] Tromp M., Ravelli A.C., Meray N., Reitsma J.B., Bonsel G.J. (2008). An efficient validation method of probabilistic record linkage including readmissions and twins. Methods Inf. Med..

[21] Stichting Perinatale Registratie Nederland (2013).

[22] Offerhaus P.M., de J.A., vdP-dB K.M., Hukkelhoven C.W., Scheepers P.L., Lagro-Janssen A.L. (2014). Change in primary midwife-led care in The Netherlands in 2000-2008: a descriptive study of caesarean sections and other interventions among 789,795 low risk births. Midwifery.

[23] Commissie Verloskunde van het College voor zorgverzekeringen (2003).

[24] Schuurhuis A., Roumen F.J., De Boer J.B. (2009). [Practice guideline 'Pharmaceutical pain treatment during labour'; the woman's request is sufficient indication]. Ned. Tijdschr. Geneeskd..

[25] Pergialiotis V., Vlachos D., Protopapas A., Pappa K., Vlachos G. (2014). Risk factors for severe perineal lacerations during childbirth. Int. J. Gynaecol. Obstet..

[26] Barba M., Bernasconi D.P., Manodoro S., Frigerio M. (2022). Risk factors for obstetric anal sphincter injury recurrence: a systematic review and meta-analysis. Int. J. Gynaecol. Obstet..

[27] Brown J., Kapurubandara S., Gibbs E., King J. (2018). The Great Divide: Country of birth as a risk factor for obstetric anal sphincter injuries. Aust. N. Z. J. Obstet. Gynaecol..

[28] Marrie R.A., Dawson N.V., Garland A. (2009). Quantile regression and restricted cubic splines are useful for exploring relationships between continuous variables. J. Clin. Epidemiol..

[29] O'Leary B.D., Ciprike V. (2020). Anal sphincter injury associated with shoulder dystocia. J. Matern. Fetal Neonatal Med..

[30] Mizrachi Y., Leytes S., Levy M., Hiaev Z., Ginath S., Bar J. (2017). Does midwife experience affect the rate of severe perineal tears?. Birth.

[31] Dencker A., Smith V., McCann C., Begley C. (2017). Midwife-led maternity care in Ireland - a retrospective cohort study. BMC Pregnancy Childbirth.

[32] O'Leary B.D., Ciprike V. (2020). Are women attending a midwifery-led birthing center at increased risk of anal sphincter injury?. Int. Urogynecol. J..

[33] Bodner-Adler B., Kimberger O., Griebaum J., Husslein P., Bodner K. (2017). A ten-year study of midwife-led care at an Austrian tertiary care center: a retrospective analysis with special consideration of perineal trauma. BMC Pregnancy Childbirth.

[34] Martin-Arribas A., Escuriet R., Borràs-Santos A., Vila-Candel R., González-Blázquez C. (2022). A comparison between midwifery and obstetric care at birth in Spain: across-sectional study of perinatal outcomes. Int. J. Nurs. Stud..

[35] Myrick T.G., Sandri K.J. (2018). Epidural analgesia and any vaginal laceration. J. Am. Board Fam. Med..

[36] Hauck Y.L., Lewis L., Nathan E.A., White C., Doherty D.A. (2015). Risk factors for severe perineal trauma during vaginal childbirth: a Western Australian retrospective cohort study. Women Birth.

[37] Turner J., Flatley C., Kumar S. (2020). Epidural use in labour is not associated with an increased risk of maternal or neonatal morbidity when the second stage is prolonged. Aust. N. Z. J. Obstet. Gynaecol..

[38] Maimburg R.D., De Vries R. (2019). Coaching a slow birth with the woman in an empowered position may be less harmful than routine hands-on practice to protect against severe tears in birth – a discussion paper. Sex. Reproduct. Healthc..

[39] Jiang H., Qian X., Carroli G., Garner P. (2017). Selective versus routine use of episiotomy for vaginal birth. Cochrane Database Syst. Rev..

[40] Ejegård H., Ryding E.L., Sjogren B. (2008). Sexuality after delivery with episiotomy: a long-term follow-up. Gynecol. Obstet. Invest..

[41] O'Malley D., Higgins A., Smith V. (2015). Postpartum sexual health: a principle-based concept analysis. J. Adv. Nurs..

[42] Aasheim V., Nilsen A.B.V., Reinar L.M., Lukasse M. (2017). Perineal techniques during the second stage of labour for reducing perineal trauma. Cochrane Database Syst. Rev..

[43] Magoga G., Saccone G., Al-Kouatly H.B., Dahlen G.H., Thornton C., Akbarzadeh M. (2019). Warm perineal compresses during the second stage of labor for reducing perineal trauma: a meta-analysis. Eur. J. Obstet. Gynecol. Reprod. Biol..

[44] Edqvist M., Dahlen H.G., Haggsgard C., Tern H., Angeby K., Teleman P. (2022). The effect of two midwives during the second stage of labour to reduce severe perineal trauma (Oneplus): a multicentre, randomised controlled trial in Sweden. Lancet.

